# Endoscopy for T10 nerve sheath tumor

**DOI:** 10.3171/2024.1.FOCVID23214

**Published:** 2024-04-01

**Authors:** Sivashanmugam Dhandapani, Chandrashekhar Gendle

**Affiliations:** Department of Neurosurgery, Postgraduate Institute of Medical Education & Research (PGIMER), Chandigarh, India

**Keywords:** minimally invasive, endoscopy, IDEM, intradural extramedullary, nerve sheath tumor, schwannoma

## Abstract

Minimally invasive surgery (MIS) is increasingly being adopted for spinal intradural tumors. Through the use of conventional microscopy or exoscopy for large lobulated nerve sheath tumors, the posterior root attachment is often visualized only after mobilizing the tumor. Here, the authors describe the utility of angled endoscopy with its panoramic view for a T10 nerve sheath tumor. Gross-total extracapsular excision was achieved utilizing a minimally invasive right paraspinous approach, fenestration, lateral durotomy, sliding delivery of the tumor, sharp dissection of radicular attachments under neuromonitoring, and dural closure with oblique clips. Angled endoscopes help visualize the attachments behind large multilobulated tumors and confirm the totality of excision.

The video can be found here: https://stream.cadmore.media/r10.3171/2024.1.FOCVID23214

## Transcript

### 0:26

Minimally invasive surgery is increasingly being adopted for spinal intradural tumors. Large IDEMs may impede the visualization of posterior attachments, and diligence is needed for the total excision of multilobulated tumors without causing neural deficits.^1^

### 0:48

A 28-year-old male patient presented with right-sided flank pain for 8 months and mild right-sided spasticity for a month. MRI of the thoracolumbar spine revealed a T10 intradural extramedullary tumor suggestive of a nerve sheath tumor toward the right.

### 1:11

The minimally invasive endoscopic approach was chosen because of the minimal disruption of the spine and soft tissues, and the panoramic view and angled optics provided by the endoscope.

### 1:25

In the prone position, the T10 level was identified using x-ray localization, and a right paramedian incision of 3 cm was given. The paraspinal muscles were retracted laterally to expose the T10 lamina, and a minimally invasive spine retractor was placed.

### 1:47

Using endoscopes of 4-mm diameter and 18-cm length for illumination and view, right-sided fenestration was carried out with a high-speed drill and rongeurs. As the tumor did not have a foraminal extension, medial facetectomy was not necessary.

### 2:08

A lateral longitudinal durotomy was done. Delineation of the extra-arachnoidal tumor plane was accomplished with the help of a dissector, using the sliding delivery technique,^2^ the tumor was gently rolled outside. Under neuromonitoring, the nonmotor nerve root entering the tumor was coagulated and disconnected.

### 2:45

By rotating the angle of the endoscope, the cord can be appreciated, with no evidence of residual tumor. The angled optics can be especially helpful in case of multilobulated tumors if a piece gets disconnected and becomes inconspicuous.^2,3^ Using microscissors, the tumor was dissected off the rootlets and was delivered en masse.

### 3:17

The operative area was irrigated with lukewarm isotonic fluid. The dura was closed with small titanium clips applied in an oblique manner using an angled applicator to lessen the chances of dural stricture. It was supplemented with multilayered soft-tissue closure. The surgery was around 110 minutes’ duration, and the blood loss was minimal.

### 3:45

The key surgical steps were a minimally invasive paraspinous approach, fenestration of the right lamina, lateral longitudinal durotomy, extracapsular delivery, sharp dissection of rootlets, and dural closure with oblique clips.

### 4:05

The patient recovered fully, with no deficit or CSF leak. The histopathology was schwannoma grade 1. Postoperative CT scan shows the extent of bony removal. Contrast MRI showed no evidence of residual tumor.

### 4:25

This minimally invasive endoscopic approach with fenestration of lamina and sliding delivery technique seems safe and effective for a T10 nerve sheath tumor.

## Disclosures

The authors report no conflict of interest concerning the materials or methods used in this study or the findings specified in this publication.

## Author Contributions

Primary surgeon: Dhandapani. Assistant surgeon: Gendle. Editing and drafting the video and abstract: Dhandapani. Reviewed submitted version of the work: Dhandapani. Approved the final version of the work on behalf of both authors: Dhandapani. Supervision: Dhandapani.

## Supplemental Information

### Patient Informed Consent

The necessary patient informed consent was obtained in this study.
